# Distribution of polyphenolic compounds, antioxidant potential, and free amino acids in *Ziziphus* fruits extract; a study for determining the influence of wider geography

**DOI:** 10.1002/fsn3.2726

**Published:** 2022-01-28

**Authors:** Nisar Uddin, Noor Muhammad, Mohammad Nisar, Aisha, Niaz Ali, Riaz Ullah, Essam A. Ali, Azhar Abbas Khan, Inayat Ur Rahman, Anwar Khan, Alam Zeb

**Affiliations:** ^1^ Department of Botany Hazara University Mansehra Mansehra Pakistan; ^2^ Department of Pomology College of Horticulture Hebei Agricultural University Baoding China; ^3^ Department of Botany University of Malakand Checkdara Pakistan; ^4^ Department of Chemistry University of Gujrat Gujrat Pakistan; ^5^ Department of Pharmacognosy College of Pharmacy King Saud University Riyadh Saudi Arabia; ^6^ Department of Pharmaceutical Chemistry College of Pharmacy King Saud University Riyadh Saudi Arabia; ^7^ Department of Biochemistry Hazara University Mansehra Mansehra Pakistan; ^8^ Institute of Molecular Plant Science University of Edinburgh Edinburgh UK; ^9^ Department of Microbiology BUITEMS Quetta Pakistan; ^10^ Department of Biochemistry University of Malakand KP Pakistan.

**Keywords:** antioxidative effects, ecological regions, free amino acid, functional food, phenolic compounds, *Ziziphus*

## Abstract

*Ziziphus* fruits have attracted much attention within the field of medicine due to their high potential against central nervous system disorders. Abundance of secondary metabolites and their composition is key to the pharmaceutical potential and commercial qualities of plants. The in vitro antioxidant activities of *Ziziphus nummularia* (Burm. f.) and *Ziziphus oxyphylla* Edgew fruit extract were analyzed using 2,2‐diphenil‐1‐pycrilhydrazyl (DPPH) and 2,2′‐azino‐bis (3‐ethylbenzothiazoline)‐6‐sulfonic acid (ABTS) free radical scavenging assay methods. Phenolic profiles were explored using high‐performance liquid chromatography‐diode array detector (HPLC‐DAD). The result revealed high concentration of polyphenols and their antioxidant potential. In *Z. nummularia*, the total phenolic content (TPC) (80.270 ± 0.422 μg/ml), DPPH (62.03 ± 0.98 μg/ml), ABTS (66.32 ± 0.73 μg/ml), and TFC (90.683 ± 0.274 μg/ml) were recorded. However, in *Z. oxyphylla*, DPPH and ABTS values were 60.66 ± 0.56 μg/ml and 61.55 ± 0.77 μg/ml, respectively, indicative of the impacts of climate and soil nutrients. The overall screening of phytochemicals revealed that both the *Ziziphus* species contain diverse bioactive compounds, including spinacetine‐3‐O‐(2 feruloyl glucopyranosyl)‐glucopyranoside, kaempferol‐3‐O‐glucoside‐7‐O‐glucoside, and caffeic acid; p‐hydroxybenzoyl hexose, p‐coumaric acid, salicylic acid, and ellagic acid pentoxide. Additionally, the highest concentrated amino acid noted was of Lue 0.19 g/100 g with 596.00 retention time (RT), followed by Thr>Ale>Isl>Phya>Val in *Z. nummularia*. Similarly, the highest concentration of Lue amino acid was recorded as 0.18/100 g with 564.52 RT followed by Pr>Thr>Ale>Lue>Isl>Phya>Val in all genotypes of *Z. oxyphylla*. Reporting of polyphenols rich and stable species along with identification of favorable regions of cultivation for amino acid, polyphenols, and higher antioxidant potential may lead the way for the identification of elite clones of the species as well as may result in new drug discovery.

## Graphical abstract

Fruit pulp from the *Ziziphus* species possesses nutritional and medicinal properties and this is the first study to apply HPLC‐DAD to estimate and identify highest concentrated polyphenols, amino acid, and as well as their use as antioxidant activity.

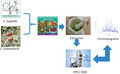

## 1 INTRODUCTION

Plants deliver a wide range of supplementary metabolites, among which phenolic compounds have gained significant attention because of their pharmacological potential and putative health benefits (Macheix et al., 2005). Secondary metabolites and bioactive compounds of plants play a key role in maintaining plants in their natural environments (Theapparat et al., 2019; Duan et al., 2019). Regulation of gene expression in response to environmental stresses results in up‐ or downregulation of the levels of the metabolites, and thus, amounts of the secondary metabolites in plants play a significant role in handling stress (Macheix et al., 2005). Furthermore, plant origin polyphenols are easily accessible and they are important antioxidants for all living beings and are being used by humans to control oxidative stresses and cardiovascular and chronic diseases (Theapparat et al., 2019; Duan et al., 2019). Accumulation of free radicals may disrupt biologically important molecules and may trigger impulses. However, polyphenols decrease the excess amounts of free radicals' generation inside the cells. These oxidative processes may lower the immunological activity and increase the chances of diabetes, infectious diseases, rheumatoid, arthritis, respiratory disorders, atherosclerosis, and a series of destructive processes due to aging, Alzheimer's disease, and schizophrenia (Mukherjee et al., 2007).

The genus *Ziziphus* is cosmopolitan in distribution and consists of about 100 species of deciduous or evergreen trees and shrubs (Chen et al., 2017; Razi et al., 2013). The genus is believed to have originated in China and South Asia and is represented widely in tropical and subtropical regions of the world including Pakistan (Razi et al., 2013). The genus *Ziziphus* has been widely used in folk and alternative medicines for treating different diseases (Adzu et al., 2001; Abdel‐Zaher et al., 2005; Nisar et al., 2007; De Omena et al., 2007; Al‐Reza et al., 2009; Bahadur et al., 2020; Ashfaq et al., 2019).

In Pakistan, *Ziziphus* is represented by six species; *Z. rugosa*, *Z. mauritiana*, *Z. nummularia*, *Z. spina‐christi*, *Z. oxyphylla*, and *Z. jujuba* (Kaleem et al., 2014; Qaiser and Nazimudin, 1984). However, two species, the *Z. nummularia* and *Z. oxyphylla*, are native and widely distributed in Districts Dir and Swat regions of Pakistan. Deep rooting and large carbohydrate reserves in its roots contribute to the strong regeneration potential of the species. The fruits of *Z. nummularia* are good sources of mineral and contain vitamin C and sugars, which contribute to cooling, astringent, appetizer, stomachic, they cure mucous, and increase biliousness effects, and dried fruits of this plant contain alkaloids, saponins, and triterpenoids (Jabeen et al., 2009). Similarly, *Z. oxyphylla* is a large shrub to a medium‐sized glabrous tree with small curved and unpaired spine along with oval‐shape edible fruits. Furthermore, *Z. oxyphylla* plant parts such as root, leaves, stem, and mostly fruits are used in folklore and traditional medicines for treating jaundice, diabetes, hypertension, as well as in gas troubles (Jan et al., 2009; Sher, 2011). All plant parts, that is, leaf, root, stem, and bark of both species, possess medicinally important properties (Dahiru & Obidoa, 2008; Dahiru et al., 2006).

High diversity of kingdom Plantae is presents in their phytochemicals too, and these phytochemicals are excellent sources of a variety of industrial and commercial applications (Khoddami et al., 2013). Indeed, several phytochemicals have been isolated from a plant that is extremely important for their pharmaceutical applications, and provide an opportunity for novel drug discovery (Zeb 2015a). Polyphenolic profile of plants can be complemented as an efficient markers system along with other morphological, biochemical, or molecular markers (Macheix et al., 2005). Free radicals inside both plant and animal cells are detrimental and they affect cell division and lead to deficiency of immunological system, risk of developing cancer, diabetes, infectious diseases, rheumatoid diseases, arthritis, respiratory diseases, atherosclerosis, and a series of destructive processes due to aging, Alzheimer's disease, and schizophrenia (Temple, 2000). Phenolic and flavonoids are secondary metabolites that include anthocyanins, hydroxycinnamic acids, and flavonoids, which play a key role in nutrition, health‐promoting, and commercial properties of plants (Zeb, 2015a, 2015b). In addition, it has a wide array of important functions in plants, structural support, and water transport (Cáceres et al., 2014; Lattanzio et al., 2008). Thus, plant parts rich in phenolic or flavonoids could serve as excellent resources to cure diseases linked to oxidative stresses. However, isolation and quantification of phenolic compounds have always posed challenges (Šola et al., 2018).

Several methods are available for the determination of phenolic compounds, that is, gas chromatography (GC), high‐performance liquid chromatography (HPLC), capillary (GC), mass spectrometry (MS), etc. (Angerosa et al., 1996). Among these options, coupling of HPLC‐MS with atmospheric pressure ionization techniques, electrospray ionization (ESI), time of flight (TOF) (García‐Villalba et al., 2010; Lozano‐Sánchez et al., 2013) are powerful and suitable tools for the precise identification of natural products in crude plant extracts (Lozano‐Sánchez et al., 2013). Furthermore, the combination of both techniques LC‐NMR offers a robust and powerful separation technique of liquid chromatography with the most information‐rich spectroscopic technique (NMR) providing structure elucidation (Christophoridou et al., 2005). Over recent years, several chromatographic methods have evolved for the identification and isolation of phenolic compounds from the plant's extracts (Khoddami et al., 2013). HPLC‐diode array detection (DAD) is one the most important and main methods which is used very broadly for the identification of phenolic compounds (Tomas‐Barberan et al., 2001).

To facilitate selection of wild species that are locally acceptable and that provide benefits such as food and medicine, the main objectives of this study were as follows: (a) to identify polyphenols in these *Ziziphus* species through HPLC‐DAD, (b) to determine the total phenolic and flavonoid contents of the fruit extract, and (c) to compare the antioxidant potential and free amino acid contents of *Ziziphus* fruit extract collected from different geographical locations.

## 2 MATERIALS AND METHODS

### 2.1 Chemicals and reagents

Methanol was purchased from Sigma‐Aldrich Steinheim (Germany), Gallic acid was provided by BDH (England), Caffeic acid and ellagic acid was purchased from Tokyo Chemical Industries, Tokyo, Japan, 2,2‐diphenil‐1 pycrilhydrazyl (DPPH) from Sigma‐Aldrich, USA (Code.101341986), and 2,2′‐azino‐bis(3‐ethylbenzothiazoline)‐6‐sulfonic acid (ABTS) from Sigma‐Aldrich, USA (Code, 1001551916). All other chemicals and reagents were of analytical grades. Ultrapure deionized water and HPLC solvent were sonicated for 30 min before chromatography.

### 2.2 Plant collection

The fruits of *Ziziphus* species were collected from different areas of Districts swat and Dir (L), Khyber Pakhtunkhwa, Pakistan (Table 1). The plants were identified using the flora of Pakistan and verified using the online resource www.theplantlist.org. The voucher specimens (NU‐001‐HUP to NU‐040‐HUP) were deposited at the Herbarium of Hazara University Mansehra, Pakistan.

**TABLE 1:** Location and distribution of *Ziziphus* two species in KP, Pakistan range of latitude, longitude, and altitude

Species	Regions	Altitude	Latitude	Longitude
*Z. nummulaira* Swat region	Barikot	783m	72°10′33.12″E	34°41′44.70″N
	Seghram	1013m	72°31′28.34″E	34°44′01.47″N
*Z. oxyphylla* Swat region	Kotlai	903m	72°30′47.33″E	34°39′13.64″N
	Sogalai	1015m	72°14′05.23″E	34°45′13.28″N
*Z. nummulaira* Dir L, region	Ghoraghat	726m	72°05′02.11″E	34°40′12.27″N
	Gullabad	709m	72°01′43.45″E	34°39′47.56″N
*Z. oxyphylla* Dir L, region	Chekdra hill	800m	72°05′44.00″E	34°40′10.27″N
	Gull muqam	768m	72°01′44.45″E	34°39′14.27″N

Note

All 40 genotypes were collected from these 08 sites of the two districts.

### 2.3 Soil analysis

During 2016, a total of 40 soil samples (three replicates each) were collected randomly from eight regions of the study area (Table 1). Samples were collected from 30 to 35 cm depth, transferred into clean polyethylene bags, and were analyzed in the Soil Lab at the Agricultural Research Institute (North) Mingora Swat, KP, Pakistan. The soil samples were air‐dried, converted into powder form by hand, passed through a sieve <2.00 mm, and stored in a polyethylene bag until they were ready for analysis. Briefly, a 0.5 g dried powdered soil sample was taken into a 50‐ml conical flask, and 15 ml of aqua‐regia (nitric acid [HNO_3_]), sulfuric acid (H_2_ SO_4_), and perchloric acid (HCLO_4_) were added to it in the ratio of 5:1:1. Samples were kept overnight and then gently heated on a hot plate at 80°C until a transparent extract was obtained. The extracts were evaluated for different compounds and their concentration was determined on an atomic absorption spectrophotometer, and soil samples were determined by the diluted hydrochloric acid (HCL) method using azomethine‐H for different color measurements as described in (20) at 420 nm on a spectrophotometer. For soil moisture analysis (%), the difference between dry and wet samples was used.

$$\begin{array}{c} \%\;\text{Soil moistures}=\text{Wet weight of dry soil (g)}/\text{wet weight (g)}*100 \end{array}$$

Nitrogen (N) and phosphorus (P) amount was determined according to Bremner (1965) and the Kjedhal method (Bingham, 1949), whereas magnesium (Mg), sodium (Na), and potassium (K) were determined using a flame photometer.

### 2.4 Determination of total phenolic content (TPC)

The total phenolic content (TPC) of *Z. nummularia* and *Z. oxyphylla* was determined using the Folin–Ciocalteu (FC) method (Zeb, 2015a, 2015b), with minor modifications. A volume of 100 µl diluted extract was taken in a test tube and 500 µl of distilled water and 100 µl of FC reagent were added, mixed, and allowed to remain for 6 min. Then, 1000 µl of 7% sodium carbonate and 500 µl of distilled water were added. After 90 min, absorbance was measured at 765 nm using a UV‐Spectrophotometer (Shimadzu). The gallic acid standard curve was obtained using dilutions (31.05, 62.5, 125, 250, 500, and 1000 µg/ml) for measuring the TPC, which was expressed as mg of gallic acid equivalent per gram (mg GAE/g) of dry sample. The total phenolic activity was estimated using a standard curve and was prepared with the absorbance noted of different concentrations of the standard, the formula being *y* = 0.0342*x* + 20.0301 (*R* ^2^ = .6959), and TPC was determined and expressed as mg of gallic acid equivalent per gram (mg GAE/g) of dry sample.

### 2.5 Total flavonoid contents (TFC)

The total flavonoid content (TFC) was investigated using the aluminum chloride colorimetry method described by Kim et al. (2002). Quercetin was used as a standard and TFC was determined in milligram of quercetin equivalent (mg QE/g). The calibration curve for quercetin was obtained using different dilutions (31.05, 62.5, 125, 250, 500, and 1000 µg/ml) prepared in methanol. A total volume of 100 µl of each of these dilutions was taken and mixed with 500 µl of distilled water. Then, 100 µl of 5% sodium nitrate was mixed and allowed to remain for 6 min. Thereafter, 150 µl of 10% aluminum chloride solution was added and allowed to remain for 5 min. Finally, 200 µl of 1 M sodium hydroxide was added and absorbance was recorded at 510 nm using UV‐Spectrophotometer. TFC contents was estimated based on the standard curve which was *y* = 0.0182*x* + 9.9939 (*R* ^2^ = .9516), in mg QE/g. All results were recorded from triplicate samples.

### 2.6 DPPH radical scavenging assay

The technique of Brand‐Williams et al. (1995) was followed for 2, 2‐diphenyl‐1‐picryl‐hydrazyl‐hydrate (DPPH) assay: 2 mg DPPH was dissolved in 100 ml methanol, while for the stock solution of samples, a concentration of 1 mg/ml was mixed in methanol and diluted to concentrations of 1000, 500, 250, 125, 62.5, and 31.5 μg/ml. For each sample of 0.1 ml, a diluted solution was mixed with 3 ml of DPPH solution in methanol and incubated for 30 min at 23°C, and then the solution absorbance was measured at 517 nm. Ascorbic acid were used as a positive control. All concentration data were observed in triplicates and the data are presented as mean ± SE; the data were calculated by the following formula:

$$\begin{array}{c} \%\text{inhibition}=\text{Control Absorbance }‐\text{Sample absorbance}/\text{Control absorbance}*100 \end{array}$$

### 2.7 ABTS free radical scavenging assay

Scavenging activity of methanolic fruit extracts of both species was assessed against 2, 2‐azinobis (3‐ethylbenzthiazoline)‐6‐sulfonic acidic (ABTS), using standard assay (Re et al., 1999). A solution of ABTS (7 mM) and potassium persulfate (2.45 mM) was prepared and kept overnight in dark to deliver free radicals and the absorbance of ABTS arrangement was changed following 0.7 at 745 nm by the expansion of 50% methanol. At this point, 300 μl of samples was taken and 3 ml of ABTS solution was added to it and absorbance was estimated at 745 nm for 6 min. For positive control, ascorbic acid was used. The information was recorded in triplicate and percent ABTS free radicals scavenging potential was calculated as follows:

$$\%\;\text{ABTS scavenging activity}=\frac{\text{Control absorbance}‐\text{sample absorbance}}{\text{absorbance Control}}\times 100$$

### 2.8 Estimation of IC_50_ values

The median inhibitory concentration, that is, IC_50_ values of DPPH and ABTS, was calculated for all test samples through the MS Excel program and origin Pro 7.5 software.

### 2.9 HPLC‐DAD analyses of phenolic compounds

Polyphenolic contents were extracted from samples by using the already‐reported method (Zeb & Ullah, 2016). The pericarp was peeled, and shade dried for 20 days and ground into fine powder. About 2 kg of powder was macerated in 80% methanol and 20% distilled water and strongly shaken. The mixture was filtered after 14 days and centrifuged for 10 min at 6000 rpm. The filtrate was subjected to a rotary evaporator for 2 h and solidified (Zeb & Ullah, 2016). For the separation of the phenolic compound using an Agilent 1260, the Infinity HPLC system consists of a degasser, auto‐sampler, quaternary, and diode array detector (DAD). The separation of the phenolic compound was carried out with the help of Agilent Rapid Resolution Zorbax Eclipse plus C18 (4.6 × 100 mm, 3.5 μm) column, which was maintained at 25°C. The gradient system consists of (10:2:88) solvent A (methanol: acetic acid: deionized water) (Zeb, 2015a, 2015b). The elution program was begun with 100% A at 0 min, 85% A at 5 min, 50% A at 20 min, 30% A at 25 min, and 100% B from 30 to 40 min. The flow rate was 1 ml/min. The chromatograms were acquired utilizing 280 nm for analysis of phenolic contents. The spectra were recorded from 190 to 450 nm. The available literature was used for the identification of compounds, its retention times, and UV absorption spectra.

### 2.10 Estimation of free amino acid

Mature and fresh fruits selected for amino acid were analyzed by the methods of acid–base titration and sulfuric acid fluorenone colorimetry (Luo et al., 2009; Wang et al., 2016). One gram of dry fruit was used for the determination of total amino acid by using HPLC (Agilent1200): 6 mol/L HCL (300 ml HCL and 300 water), 0.1 mol/L (8.3 ml HCL and 1 liter water), and Na_2_CO_3_/Na2HCO_3_ (0.53 g Na_2_CO_3_ and 0.42 g Na2HCO_3_ [pH;9]) were dissolved in 10 ml distilled water, and 3 g poison was added to 10 ml CH_3_CN, and 2.5 g CH_3_COONa (1.5 triethylamine and 1170 μl CH_3_COOH) was added to 1 liter water, after which nylon filter paper was used. Ten milliliter (6 mol/L HCL) was added and placed in the oven for 24 h up to 110°C, before the oven process closes the test tube through the air‐born pump (Butan China) then place it into an electric thermostatic blast oven for 24 h. 2 ml liquid was taken and centrifuged (8000 rpm) for 6 min; the and dried by using nitrogen evaporator system and heating system for up to 90°C, then 0.1 mol/L HCL (2 ml) was added and kept into the water bath for 90 min on 90°C, and 50 μl (10%) CH_3_COOH and 550 μl water added than, filtered through a nylon filter paper for the HPLC analyses to identify the total amino acid in *Ziziphus*. Proline was used as a stander concentration of 1, 0.5, 0.4, 0.3, 0.2, and 0.1 mg/ml.

%AM (Amino acid)=(0.0001X (‐)‐0.0035)X2.

### 2.11 Statistical analysis

All data were taken in triplicates and mean ± SE values were analyzed for the variation by one‐way analysis of variance (ANOVA *α* = 0.05) and correlation among different assays was determined using Sigma Plot (Systat Software Inc., 2013). Basic statistics and estimation of IC_50_ values of DDPH and ABTS were carried out using MS Excel 2016.

## 3 RESULT AND DISCUSSION

### 3.1 Bioactive compounds in *Ziziphus* species collected from different areas

Different phenolic compounds are naturally plant‐occurring phytochemicals and are highly important, as they possess antioxidant potential to control oxidative activities/oxidative rancidity of food (Guo et al., 2020). Flavonoids are the largest groups of phenolic compounds, namely flavanols, flavones, isoflavones, flavones, and chalcones, which have shown different activities such as antioxidant, anticarcinogenic, and cardioprotective assays (Khan et al., 2011).

The current soil analysis included physical and chemical abilities of the selected soil samples profiling, soil pH, and nutrient cycles between plant species and soil, which are most important in determining the area's soil properties. In the current analysis, pH of the soil ranged from 7.4 to 8.0, which shows that there is not much variation in the pH values of different collected soil samples. The soil analysis (% clay, silt, sand, soil moisture, %N, Na, Mg, and %K) was determined (Table S1). The soil moisture and clay ranged from 34% to 91% and 8.8 to 18%, and the maximum nitrogen (N) and potassium (K) content was observed in District Swat as 0.54 + 0.7 to 0.89 + 0.2 and 1.0 + 0.2 to 3.0 + 0.16. While that of the District Dir was 0.15 + 0.09 to 0.67 + 0.10 and 1.8 + 0.24 to 3.0 + 0.23, respectively, in both *Ziziphus* species. The availability of Na% and Mg% ranged from 0.14 + 0.09 to 0.89 + 0.04 and 0.45 + 0.13 to 0.89 + 0.04 in both *Ziziphus* species, respectively (Table 2); a similar result was recorded in the previous findings (Wahab et al., 2008; Khan et al., 2011).

**TABLE 2:** Total phenolic and flavonoid content of fruits methanolic extract of *Ziziphus* species collected from the different regions of KP, Pakistan

Species	Regions	TPC (mg GAE/g)	TFC (mg QE/g)
*Z. nummularia*	Barikot	75.067 ± 0.122	89.850 ± 0.635
	Seghram	80.270 ± 0.422	88.953 ± 0.942
*Z. oxyphylla*	Kotlai	69.520 ± 0.819	90.683 ± 0.274
	Sogalai	75.303 ± 0.673	90.447 ± 0.288
*Z. nummularia*	Ghoraghat	64.083 ± 0.982	88.547 ± 0.675
	Gullabad	66.313 ± 0.518	88.793 ± 0.476
*Z. oxyphylla*	Checkdra hill	66.127 ± 0.517	87.368 ± 0.721
	Gull muqam	68.207 ± 0.573	89.231 ± 0.612

Note

Data are calculated as mean ± standard deviation (*n* = 3). Values in the same column with different superscripts are significantly different (*p* < .05).

In the current study, total phenolic and flavonoids content was determined in fruit methanolic extracts of *Ziziphus* species. Both *Ziziphus* species have significant amounts of TFC and TPC. Gallic acid and quercetin were used as a standard for the determination of TPC and TFC to construct standard collaboration curves by dilutions such as 20, 40, 60, 80, and 100 mg/ml. *Z. nummularia* plant were collected from two different regions District Swat and Dir (L) to estimation of TPC by the use of methanolic fruits extect, the results as 75.067 ± 0.122 collected from the Barikot region while other from Seghram 80.270 ± 0.422. And 69.520 ± 0.819 and 75.303 ± 0.673 were collected from District Dir lower (Ghoraghat and Gullabad (Dir (L)). *Z. oxyphylla* collected from District Swat and Dir lower was 64.083 ± 0.982 and 66.313 ± 0.518, respectively, while the TFC highest data were recorded in *Z. oxyphylla* which were 90.683 ± 0.274 and 90.447 ± 0.288; all data are presented in Table 2. The area of the collection was although different (Table 1), the geographical locations are within the altitudinal range 768 to 1015 meters. Besides, our soil analysis indicated high variation in both regions of KP, and this marked variation in the TPC or TFC may be attributed to the soil type (Table S1). Our result confirmed that the Blueberries (46.56 mg GAE/g dry extract), cranberries (22.13 mg GAE/g dry extract), and gooseberries (5.37 mg GAE/g dry extract) (Deng et al., 2015). Previously, the difference in climatic conditions, as well as soil types, has been shown to affect the composition of secondary metabolites including polyphenol and flavonoid contents (Deng et al., 2015; Ouerghemmi et al., 2016). The current results are in agreement with the results of Katalinić et al. (2010), who reported the total TPC components in different parts of the grape plants and showed that red grapes have a higher content of phenolic compounds than others, while the results of the reported study are different from those reported in the literature. Özkan et al. (2004) reported that the TPC content of the different solvents extracts was different as follows: Narice was 627.9 mg GAE/g. Özkan et al. (2004) estimated the TPC of Emir and Kalecik karasi pomace extract to be 86.8 and 96.3 mg GAE/g, respectively; these differences may be due to the different methods of extraction used and due to different geographical distribution and soil nutrients availability (Ozcan, 2006).

The role of phenolic and flavonoids, for example, protection of plants, treatment of carbohydrate absorption like diabetes, inhibits the absorption of amylase, inhibits the risk of metabolic syndrome, which are related to complications of type 2 diabetes, and mostly used against different diseases such as aging, anti‐inflammatory, antioxidant, and anti‐proliferative agents, as well as with enhancement of bile secretion, and reduce blood (Moo‐Huchin et al., 2015; Sales et al., 2012; Shukitt‐Hale et al., 2008). The current study has confirmed that the qualitative and quantitative polyphenol composition is dependent on environmental conditions (Deng et al., 2015; Moore et al., 2014; Verm & Shukla, 2015); as well, the intraregional evaluation allows noting that polyphenols are more abundant in *Z. nummularia* genotypes compared to *Z. oxyphylla* for both regions. The present finding is comparable to phenolic contents of seed extracts of date (55 mg GAE/g extract) (Deng et al., 2015), grape (35–65 mg GAE/g), jackfruit (27.7 mg GAE/g), litchi (17.9 mg GAE/g), longan (62.6 mg GAE/g), kinnow (3.68 mg GAE/g), and tamarind (94.5 mg GAE/g) at various time, temperature, and solvent combinations of extraction (Soong & Barlow, 2004). Phenolic compounds in fruits may vary, depending on some factors like cultivar, maturity stage, cultural practice, location, climate, and other factors (Shukitt‐Hale et al., 2008); for example, berries grown in colder climates contain much more phenolic compounds than those grown in milder climate (Sales et al., 2012).

### 3.2 Antioxidant potential of *Ziziphus* species

**FIGURE 1:**
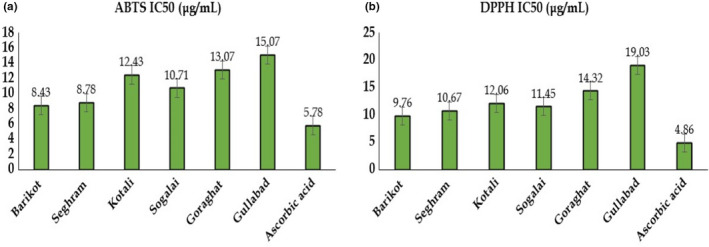
IC_50_ values of *Ziziphus* species collected from different regions of KP, Pakistan. (a) represents ABTS and (b) (DPPH)

The radical scavenging activity of plant extracts contributes to the presence of different phenolic compounds as well as to the relationship among all these compounds (Barros et al., 2010; Miguel et al., 2009). Here, not only the amount of phenolic and flavonoid content was estimated but also their antioxidant potential was assessed using ABTS and DPPH assays. The free radical scavenging activities of methanolic fruits extract from two *Ziziphus* species, *Z. numularia* and *Z. oxyphylla*, showed maximum antioxidant scavenging activity as compared to *Z. oxyphylla*. Both species of *Ziziphus* collected from different regions show the scavenging activity in the lowest concentration percent DPPH activity range of 31.25 µg/ml, which was 63.48 ± 1.43, 62.03 ± 0.98, 62.99 ± 0.30, 62.26 ± 0.34, 60.66 ± 0.56, and 59.66 ± 0.21 µg/ml. In the current result, genotypes of *Z. nummularia* collected from District Swat (Barikot and Seghram) showed the highest percent DPPH scavenging inhibition. In ABTS, the methanolic fruit extract of *Z. nummularia* and *Z. oxyphylla*, which was collected from two different regions of KP, Pakistan, shows a valuable result of all genotypes of *Z. oxyphylla* and *Z. nummularia*. The *Z. nummularia* genotypes which were collected from District Swat and Dir lower percent inhibition of ABTS free scavenging activity in lowest concentration which as 64.81 ± 1.17, 66.32 ± 0.73, 61.55 ± 0.77 and 60.45 ± 0.78, respectively. While *Z. oxyphylla* genotypes collected from District Swat, which were represented inhibition against ABTS percent inibition as 59.29 ± 0.98 and 60.17 ± 0.44, and the standard curve of both DPPH and ABTS activities while methanol was used for the blank and result was represented as median effective concentration getting by the linear regression (Table S2 and Figure S1). A substantial antioxidant activity was found which is dependent on their geographical origin and thus their different environmental features of the plants' growth condition; the antioxidant/radical scavenging capacity of such extracts could also be attributed to the presence of other bioactive components, with chemical structures different from phenols, as well as to the interactions among all these compounds (Barros et al., 2010; Miguel et al., 2009; Zietz et al., 2010). Ascorbic acid was used as a standard, showing inhibition of scavenging activity in the lowest concentration of 31.25 µg/ml against DPPH with IC_50_ value as 10.86 µg/ml and ABTS as 67.61 ± 0.71 µg/ml with IC_50_ value as 5.78 µg/ml (Figure 1); a similar result was found in previously published studies (Meda et al., 2005; Ahmad et al., 2016; Kumarasamy et al., 2007; Olajuyigbe and Afolayan, 2011).

### 3.3 Pearson's correlation analysis

Pearson's correlation analysis (*r* ^2^) among TPC, TFC, DPPH, and ABTS, soil nutrients, and dry weight is summarized in Table 3. The TFC, DPPH, and ABTS have been found to show a positive correlation of 0.894, 0.989, and 0.899 with TPC, respectively, whereas, in case of TFC, correlation values of 0.876 and 0.983 were observed. Furthermore, The TPC and TFC also showed positive correlation with respect to each other (0.876). Similar results were found in a previous research which reported that higher phenolic content contributed to higher antioxidant activity and a linear correlation was observed between bioactive compounds and antioxidant activity (Abbaszadeh et al., 2013; Liu et al., 2009; Sharma & Cannoo, 2016).

**TABLE 3:** The antioxidant activity of fruit methanolic extract of *Ziziphus* species *Z. nummularia* and *Z. oxyphlla*

Genotypes	Location	(μg/ml)	% ABTS	% DPPH
			Mean ± SEM	Mean ± SEM
*Z. nummularia* District Swat	Barikot	1000	86.54 ± 0.17	90.05 ± 1.02
		500	83.62 ± 0.48	87.05 ± 0.89
		250	76.83 ± 1.31	80.43 ± 0.43
		125	74.71 ± 0.94	74.31 ± 0.54
		62.5	68.71 ± 0.77	67.29 ± 0.78
		31.25	64.81 ± 1.17	63.48 ± 1.43
	Seghram	1000	87.67 ± 0.19	90.89 ± 0.89
		500	85.02 ± 0.16	83.29 ± 0.67
		250	78.23 ± 0.38	80.03 ± 0.54
		125	71.49 ± 0.42	75.97 ± 1.12
		62.5	68.78 ± 0.93	73.70 ± 0.41
		31.25	66.32 ± 0.73	62.03 ± 0.98
*Z. nummularia* District Dir Lower	Ghoraghat	1000	91.12 ± 0.73	93.60 ± 3.11
		500	85.15 ± 0.72	87.75 ± 0.56
		250	79.93 ± 0.16	79.75 ± 0.61
		125	71.21 ± 0.28	73.25 ± 1.05
		62.5	64.34 ± 1.34	68.23 ± 0.42
		31.25	59.29 ± 0.98	62.99 ± 0.30
	Gullabad	1000	92.11 ± 0.83	91.89 ± 1.09
		500	89.36 ± 0.71	86.09 ± 0.13
		250	80.41 ± 0.77	81.98 ± 1.05
		125	71.21 ± 0.37	73.72 ± 0.16
		62.5	65.11 ± 0.98	68.27 ± 0.17
		31.25	60.17 ± 0.44	62.26 ± 0.34
*Z. oxyphylla* District Swat	Kotlai	1000	91.03 ± 0.36	91.83 ± 0.98
		500	87.34 ± 0.34	86.12 ± 0.14
		250	81.43 ± 0.43	79.98 ± 1.01
		125	76.51 ± 0.61	73.71 ± 0.13
		62.5	67.19 ± 0.77	64.29 ± 1.03
		31.25	61.55 ± 0.77	60.66 ± 0.56
	Sogalai	1000	89.58 ± 0.44	89.68 ± 0.96
		500	85.34 ± 0.21	82.67 ± 0.14
		250	79.16 ± 0.42	75.34 ± 1.21
		125	72.51 ± 0.61	68.43 ± 0.89
		62.5	65.19 ± 0.87	63.11 ± 0.18
		31.25	60.45 ± 0.78	59.66 ± 0.21
*Z. oxyphylla* District Dir Lower	Checkdra hill	1000	89.21 ± 0.53	93.60 ± 3.11
		500	84.14 ± 0.70	87.75 ± 0.56
		250	76.93 ± 0.62	87.75 ± 0.56
		125	71.21 ± 0.28	73.25 ± 1.05
		62.5	67.33 ± 0.89	68.23 ± 0.42
		31.25	59.09 ± 1.19	62.99 ± 0.30
	Gull muqam	1000	88.22 ± 0.73	89.09 ± 1.08
		500	83.46 ± 0.75	86.13 ± 0.17
		250	79.50 ± 0.77	80.89 ± 1.07
		125	72.00 ± 0.67	74.62 ± 0.24
		62.5	64.76 ± 0.98	69.17 ± 0.20
		31.25	59.28 ± 0.44	63.24 ± 0.46
Ascorbic acid		1000	95.32 ± 1.54	97.77 ± 0.28
		500	89.54 ± 0.47	91.67 ± 0.72
		250	82.36 ± 0.44	85.96 ± 0.29
		125	76.91 ± 0.49	80.10 ± 0.57
		62.5	71.74 ± 0.82	73.48 ± 0.76
		31.25	67.61 ± 0.71	70.70 ± 0.47

Note

Data are calculated as mean ± standard deviation (*n* = 3). Values in the same column with different superscripts are significantly different (*p* < .05).

### 3.4 Polyphenols identification by HPLC‐DAD

**FIGURE 2:**
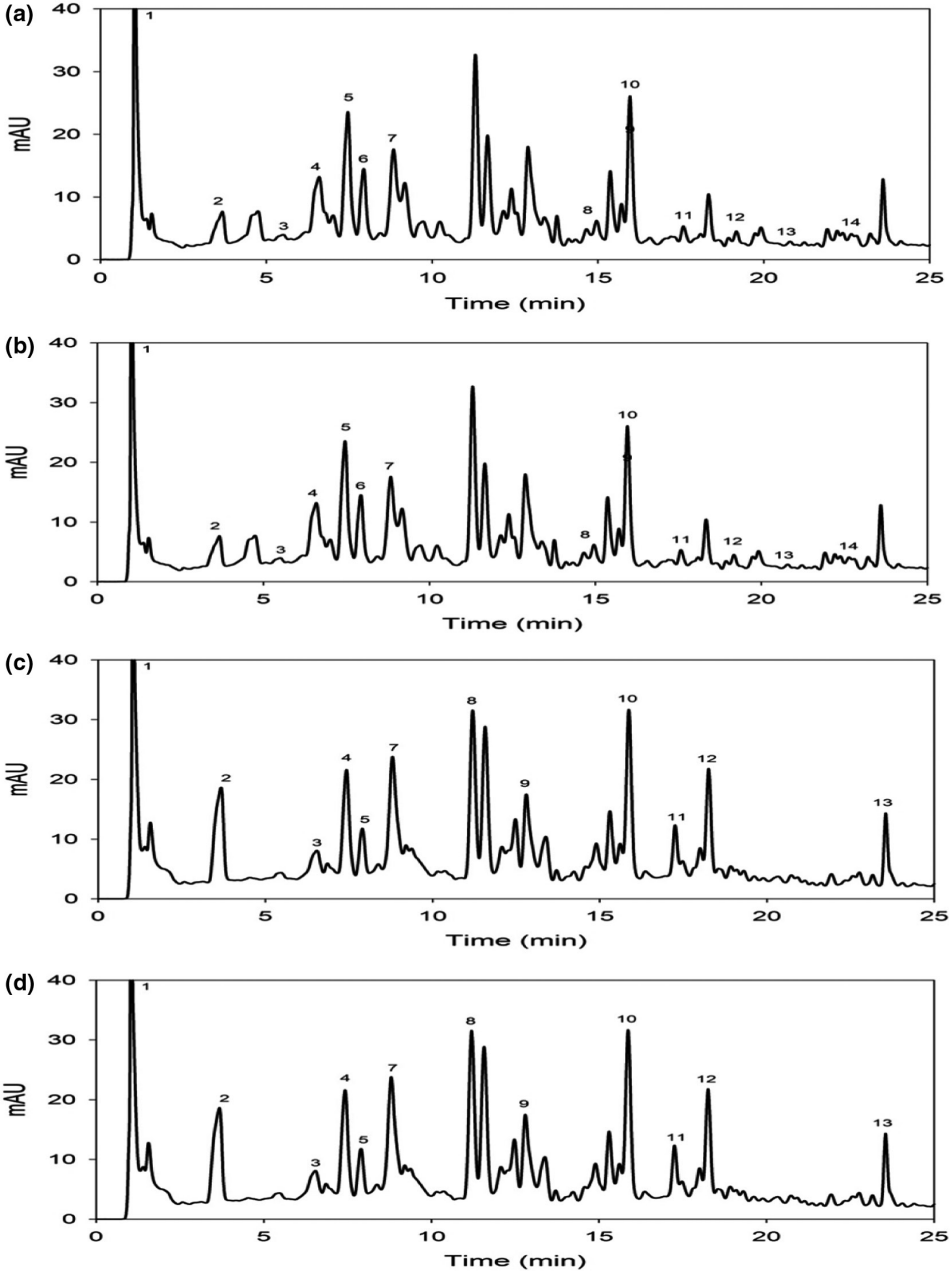
HPLC‐DAD chromatograms of methanolic extracts from the fruits of (a and b) *Z. nummualaria* and (c and d) *Z. oxyphylla*. Retention time and peak area are shown in Table 6

Fruits are rich sources of bioactive constituents such as antioxidants which can neutralize lipid free radicals or inhibit the accumulation of free radicals, and thus suppress the accumulation of volatile products from hydroperoxides: for example, aldehydes and ketones, which give unpleasant odors and flavors that may cause rancidity to lipid‐containing foods (Leal et al., 2008; Santos et al., 2014). They are also known to exhibit antiviral, antibacterial, antiallergenic, and anti‐inflammatory activities, as well as reduce the risk of heart disease, cancer, and diabetes (Leal et al., 2008; Santos et al., 2014). The chromatographic separations of individual polyphenols in standard and methanol extracts of *Ziziphus* species are depicted in Figure 2. The concentration of every phenolic compound identified in methanolic extracts was measured from the corresponding calibration curve of the methanolic extracts of *Ziziphus* species.

A total of 14 polyphenolic compounds were recorded from *Z. nummularia* collected from Swat and Dir (L), while *Z. oxyphylla* had a total of 13 compounds identified by the HPLC‐DAD spectra, their retention time and reported peak area of the compounds standards were identified, and the results are presented in (Tables 4 and 5). The gallic acid was used the first to elute and identify a compound as 1.0 min. or all genotypes for the quantification and identification of phenolic compounds using the and measure their standard calibration, absorption spectra, and retention time. The data are presented in Tables 4 and 6. The current investigated compounds as shown in Figure 2, followed by gallic acid derivative, cinnamic acid derivative, and ellagic acid derivative, were identified as compounds 2, 3, and 4, and their peak and retention time was 3.6, 5.3, and 6.5 min, respectively. The current results are similar with those reported by the authors using HPLC and MS/GC methods, which were correlated with the fifth compound, as 5‐O‐caffeoylquinic acid, caffeic acid hexoside, caffeic acid, quercetin 3‐rutinoside, quercetin‐3‐galactoside, kaempferol‐3‐O‐glucoside‐7‐O‐glucoside, quercetin derivative, spinacetine‐3‐O‐(2‐feruloyl glucopyranosyl)‐glucopyranoside, and proanthocyanidin B1 with retention time 22.9 min were examined (Table 4), while the *Z. oxyphylla* has some differences, which as collected from two different areas District Swat, the data are characterized in Table 1.

**TABLE 4:** Identification and composition of polyphenols compounds in *Z. nummularia* collected from swat district (Barikot and Seghram) and district Dir (Ghoraghat and Gullabad) regions samples using HPLC‐DAD

No.	Phenolic compound	Rt (min)	HPLC–DAD *λ*_max_ (nm)		Rt (min)	%Mass and area	References
		Dir (Ghoraghat and Gullabad)		Swat (Barikot and Seghram)			
1	Gallic acid	1	271	271	1	7.113	Santos et al. (2014)
2	Gallic acid derivative	3.6	279	279	3.6	7.323	Santos et al. (2014)
3	Cinamic acid derivative	5.3	320, 280	320, 280	5.3	8.399	Santos et al. (2014)
4	Ellagic acid derivative	6.5	265, 298sh	265, 298sh	6.5	6.955	Santos et al. (2014)
5	5‐O‐Caffeoylquinic acid	7.4	326, 244	326, 244	7.4	8.556	Santos et al. (2014)
6	Caffeic acid hexoside	7.8	290, 293	290, 293	7.8	7.612	Fischer et al. (2011)
7	Caffeic acid	8.8	323, 238	323, 238	8.8	8.478	Fischer et al. (2011)
8	Quercetin 3‐rutinoside	14.6	258,355	258,355	14.6	6.772	Jang et al. (2018)
9	Qurecetin‐3‐O‐galactoside	15.8	355, 255	355, 255	15.8	9.318	Santos et al. (2014)
10	Luteolin‐7‐O‐glucoside	16.2	350, 268, 255	350, 268, 255	16.2	9.186	Santos et al. (2014)
11	Kaempferol‐3‐O‐glucoside‐7‐O‐glucoside	17.2	342, 266	342, 266	17.2	8.976	Aaby et al. (2007)
12	Qurecetin derivative	19	324, 267	324, 267	19	8.504	Santos et al. (2014)
13	Spinacetine‐3‐O‐(2‐feruloylglucopyranosyl)‐glucopyranoside	20.8	358, 313, 256	358, 313, 256	20.8	8.565	Santos et al. (2014)
14	Proanthocyanidin B1	22.9	310, 286	310, 286	22.9	9.396	Aaby et al. (2007)

**TABLE 5:** Identification and composition of polyphenolic compounds in *Z. oxyphylla* collected from Swat (Kotlai and Sogalai) and Dir lower (Checkdara hill and Gull muqam) regions samples using HPLC‐DAD

No.	Rt (min)	Phenolic compound	HPLC–DAD *λ*_max_ (nm)	Rt (min)	HPLC–DAD *λ*_max_ (nm)	References
	Swat district (Kotlai and Sogalai)			Dir (Checkdara hill and Gull muqam)		
1	1.0	Gallic acid	271	1.0	271	Santos et al. (2014)
2	3.6	Gallic acid derivative	279	3.6	279	Santos et al. (2014)
3	6.5	Ellagic acid derivative	265, 298sh	6.5	265, 298sh	Santos et al. (2014)
4	7.4	p‐Hydroxybenzoylhexose	262	7.4	262	Santos et al. (2014)
5	7.8	Caffeic acid hex	290, 293	7.8	290, 293	Fischer et al. (2011)
6	8.8	p‐Coumaric Acid	310, 230	8.8	310, 230	Santos et al. (2014)
7	11.1	5‐O‐p‐Coumaroylquinic acid	310, 232	11.1	310, 232	Santos et al. (2014)
8	11.5	Salicylic acid	302, 236	11.5	302, 236	Santos et al. (2014)
9	12.8	Luteolin‐7‐O‐glucoside	350, 268, 255	12.8	350, 268, 255	Santos et al. (2014)
10	15.8	Qurecetin‐3‐D‐galactoside	355, 255	15.8	355, 255	Santos et al. (2014)
11	17.2	Quercetin 3‐glucoside	356, 256	17.2	356, 256	Santos et al. (2014)
12	18.2	Ellagic acid pentoside	360, 254	18.2	360, 254	Santos et al. (2014)
13	23.5	Proanthocyanidin B1	310, 286	23.5	310, 286	Aaby et al. (2007)
14	22.9	Proanthocyanidin B1	310, 286	310, 286	22.9	Aaby et al. (2007)

**TABLE 6:** Polyphenols, location, and their concentration in mg/100 g of *Z. nummularia* and *Z. oxyphylla*

Compounds	Concentration mg/100 g								
*Z. nummularia*	Swat		Dir lower		*Z. oxyphylla*	Swat		Dir lower	
Barikot + Seghram			Ghoraghat + Gullabad		Kotlai + Sogalai			Checkdara hill + Gull muqam	
	Mean ± SED					Mean ± SED			
Gallic acid	11.9 ± 0.3	15.3 ± 0.5	1.98 ± 0.01	7.91 ± 0.1	Gallic acid	9.25 ± 0.2	4.78 ± 0.1	5.25 ± 0.2	4.90 ± 0.3
Gallic acid derivative	1.81 ± 0.01	1.87 ± 0.1	1.66 ± 0.01	0.678 ± 0.01	Gallic acid derivative	0.398 ± 0.01	0.576 ± 0.03	0.98 ± 0.01	0.76 ± 0.03
Cinnamic acid derivative	23.0 ± 0.5	23.2 ± 0.3	0.928 ± 0.02	0.666 ± 0.01	Ellagic acid derivative	1.91 ± 0.05	2.93 ± 0.05	3.00 ± 0.05	3.00 ± 0.05
Ellagic acid derivative	15.2 ± 0.2	11.3 ± 0.2	2.27 ± 0.1	2.52 ± 0.03	P Hydroxybenzoylhexose	3.66 ± 0.1	2.91 ± 0.1	6.56 ± 0.1	3.01 ± 0.1
5‐O‐Caffeoylquinic acid	2.15 ± 0.1	2.18 ± 0.1	3.32 ± 0.1	3.30 ± 0.05	Caffeic acid hex	1.41 ± 0.07	0.776 ± 0.04	2.41 ± 0.07	1.01 ± 0.04
Caffeic acid hexoside	2.95 ± 0.1	3.31 ± 0.1	1.97 ± 0.02	1.60 ± 0.1	p‐Coumaric Acid	4.39 ± 0.1	1.54 ± 0.07	4.39 ± 0.1	2.50 ± 0.07
Caffeic acid	6.43 ± 0.2	6.69 ± 0.2	1.89 ± 0.04	2.34 ± 0.1	5‐O‐p‐Coumaroylquinic acid	4.69 ± 0.1	5.38 ± 0.1	7.69 ± 0.1	5.90 ± 0.02
Quercetin 3‐O‐rutinoside	44.6 ± 1.1	49.5 ± 1.3	1.86 ± 0.1	0.471 ± 0.02	Salicylic acid	3.98 ± 0.06	3.23 ± 0.1	3.89 ± 0.06	3.23 ± 0.01
Qurecetin‐3‐O‐galactoside	30.9 ± 0.8	32.6 ± 1.8	3.96 ± 0.05	0.735 ± 0.03	Luteolin‐7‐O‐glucoside	6.21 ± 0.2	4.45 ± 0.2	5.21 ± 0.2	5.00 ± 0.2
Luteolin‐7‐O‐glucoside	11.4 ± 0.5	12.9 ± 0.3	0.557 ± 0.01	2.54 ± 0.01	Qurecetin‐3‐D‐galactoside	10.5 ± 0.3	3.73 ± 0.03	14.5 ± 0.3	3.0.56 ± 0.13
Kaempferol‐3‐O‐glucoside‐7‐O‐glucoside	13.8 ± 0.7	15.5 ± 0.4	0.852 ± 0.02	0.349 ± 0.02	Quercetin 3‐glucoside	3.34 ± 0.04	1.29 ± 0.01	5.34 ± 0.04	1.52 ± 0.11
Qurecetin derivative	15.8 ± 0.9	12.1 ± 0.6	0.647 ± 0.01	0.181 ± 0.01	Ellagic acid pentoside	6.00 ± 0.1	1.51 ± 0.02	6.00 ± 0.1	2.78 ± 0.02
Spinacetine‐3‐O‐(2‐feruloylglucopyranosyl)‐glucopyranoside	11.4 ± 0.3	6.95 ± 0.3	0.287 ± 0.01	0.699 ± 0.01	Proanthocyanidin B1	4.07 ± 0.1	1.47 ± 0.07	0.07 ± 0.1	2.10 ± 0.07
Proanthocyanidin B1	2.95 ± 0.2	3.75 ± 0.1	0.345 ± 0.01	0.369 ± 0.01					

Identification and composition of polyphenols compounds were performed in *Z. nummularia* collected from Swat District (Barikot and Seghram) and District Dir (Ghoraghat and Gullabad) regions samples using HPLC‐DA. Gallic acid, gallic acid derivative and ellagic acid derivative were identified for the frist time into differnet time scale which as 1.0, 3.6, and 6.5 min. the p‐Hydroxybenzoyl hexose, retention time as 7.4, min followed by p‐coumaric acid and 5‐O‐p‐coumaroylquinic acid which as 8.8 and 11.1 min. which is differentiate *Z. oxyphylla* species from the other species. And the proanthocyanidin B1 23.5 as a retention time was noted (Table 5). Similarly, identification and composition of polyphenolic compounds were done in *Z. oxyphylla* collected from Swat (Kotlai and Sogalai) and Dir (L) (Checkdara hill and Gull muqam regions samples using HPLC‐DAD (Table 5)).

The general classification was noted using Shi et al. (2003). The phenolic compounds in different plants may be divided into two categories, phenolic acid and flavonoids, which were noted as cinnamic acids (caffeic acid, quercetin 3‐rutinoside, quercetin‐3‐galactoside, and kaempferol‐3‐O‐glucoside‐7‐O‐glucoside) and benzoic acids (p‐hydroxybenzoic, protocatechuic, vanillic, and gallic acids) (Ozcan, 2006). Even though these results were obtained from different plants which were similar we recorded in the current result and identified the correlation with our polyphenols. *Z. nummularia* collected from both districts had very closely related compounds and also contained a high number of phenols, that is, caffeic acid, quercetin 3‐rutinoside, quercetin‐3‐galactoside, kaempferol‐3‐O‐glucoside‐7‐O‐glucoside, and quercetin derivative presented in Table 6. A similar result was mentioned in Santos et al. (2014) and Jan et al. (2018). *Brassica* family has also been identified by a different phenolic which is like our results, that is, quercetin‐3‐O‐triglucoside (Schmidt et al., 2010). Quercetin derivative and quercetin are found in protecting the cells from different cytotoxicities and hydrogen peroxide. A similar observation was recorded by Zeb (2015a).

**FIGURE 3:**
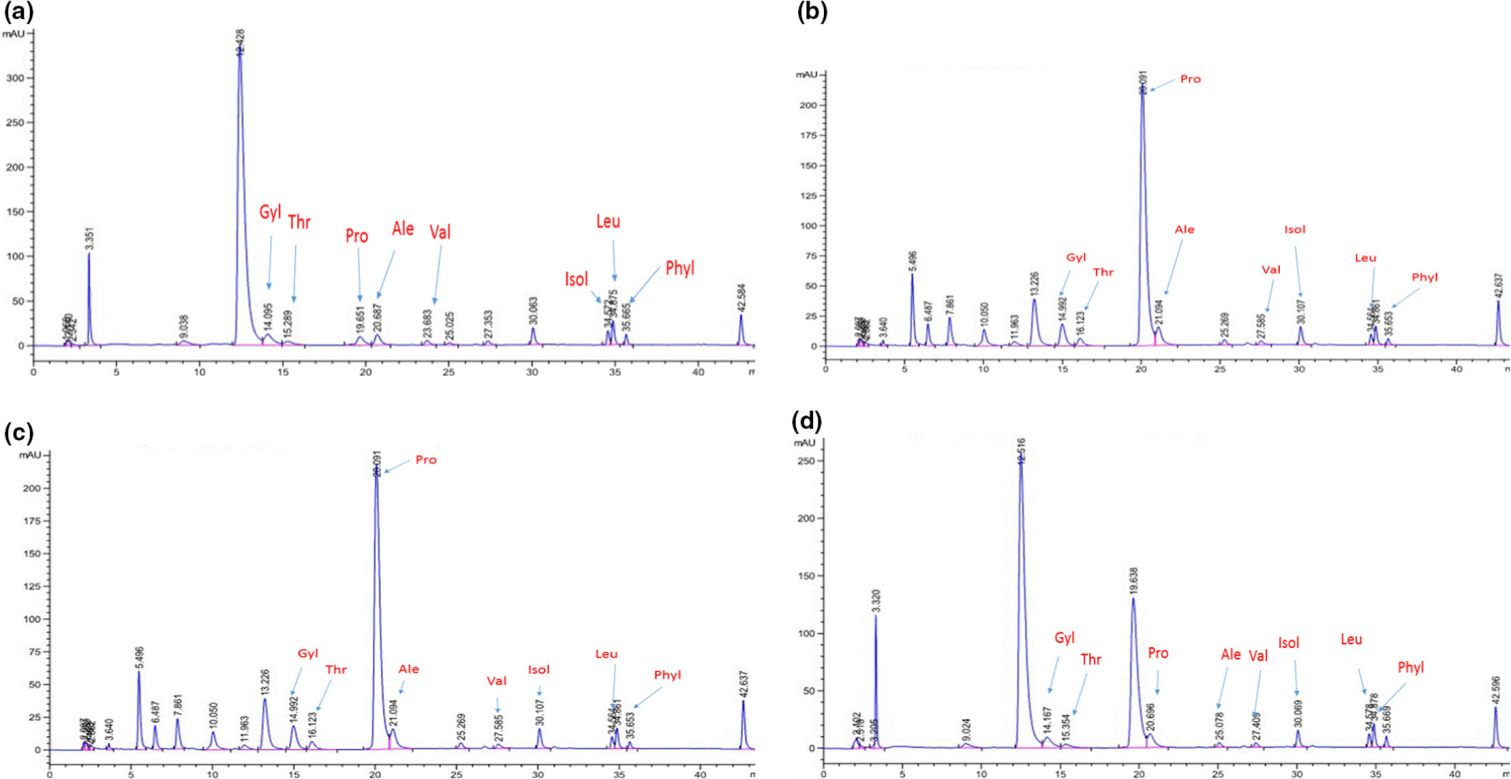
Peaks of amino acids and their retention time in samples of *Ziziphus* species. (a) design for *Z. nummularia* Swat, Barikot; Seghram, (b) for *Z. nummularia* Dir L, Ghoraghat; Gull muqam, (c) for *Z. oxyphylla* Swat, Kotlai; Sogalai, and (d) for *Z. oxyphylla* Dir L, Gullabad; Checkdra hill

Thus, *Ziziphus* species have large amounts of quercetin and their derivatives which help in antioxidant activities (Lu et al., 2011; Mohammed et al., 2013; Pontis et al., 2014; Sohaib et al., 2015; Zeb, 2015a, 2015b). Many variations were recorded in the composition of the compounds among the *Ziziphus* species the chromatogram were represented in (Figure 3). To the best of our abilities, the current investigation is the first report of its kind. These metabolites were isolated and identified from fruits of the *Ziziphus* species by HPLC‐DAD and mainly detected from methanolic extracts. The result reveals that the qualities and quantities of the compoundes/polyphnoles depend on environmental condition which affect their quantities and composition of compounds and thereby the antioxidant potential effects on improving health, food to prevent or delay the oxidation of food, initiated by free radicals formed during their exposure to environmental factors such as air, light, and temperature (Hraš et al., 2000; Deng et al., 2015; Verm & Shukla, 2015; Moore et al., 2014; Siracusa & Ruberto, 2014; Macheix et al., 2005; Olajuyigbe and Afolayan, 2011).

Total polyphenols were measured by HPLC‐DAD; the methanolic extracts range from 0.287 to 49.5 mg/100 g. The highest concentration of phenolic compounds was recorded in *Z. nummularia* from the Swat region and Dir lower region. And the lowest concentration was noted in *Z. oxyphylla*. The highest concentration of phenolic compounds was observed in *Z. nummulara*, which was 49.5 ± 1.3 and 32.6 ± 1.8 mg/g, respectively. In the samples which were collected from Swat, the compounds ranged from 44.6 ± 1.1 and 30.9 ± 0.8 mg/100 g. While lowest polyphenol compound concentrations were noted in *Z. oxyphylla* collected from Swat region with a high concentration range of 7.91 ± 0.1 mg/100 g, the lowest range was 0.287 ± 0.01 (Ouerghemmi et al., 2016; Santos et al., 2014; Zeb, 2015a, 2015b). The current work for the first time identified phenolic compounds through HPLC‐DAD from the fruit of *Z. nummularia* and *Z. oxyphylla* collected from Swat and Dir regions. Furthermore, the methanolic extract was used for to identified different compounds such as cinnamic acid derivative, ellagic acid derivative, 5‐O‐caffeoylquinic acid, caffeic acid hexoside, luteolin‐7‐O‐glucoside, spinacetine‐3‐O‐(2‐feruloyl glucopyranosyl)‐glucopyranoside, and proanthocyanidin B, the quercetin 3‐O‐rutinoside, quercetin‐3‐O‐galactoside, and kaempferol‐3‐O‐glucoside‐7‐O‐glucoside are major and important compounds with representing with the high concentration data are presented in (Table 6).

Furthermore, the free radical scavenging activity measured by an individual activity is strictly dependent on the climatic conditions which are demonstrated in this assay. The antioxidant activity of fruit methanolic extracts of *Ziziphus* species which contributed to the control of free radical scavenging was due to high amount of phenolic and flavonoids (Biological compounds) found in fruit extracts of *Ziziphus* species *Z. nummularia* and *Z. oxyphylla*. Unique types of compounds from the other *Ziziphus* such as spinacetine‐3‐O‐(2 feruloyl glucopyranosyl)‐glucopyranoside, kaempferol‐3‐O‐glucoside‐7‐O‐glucoside, and caffeic acid were found only in *Z. nummularia* which are used for the cure of different diseases such as inhibiting free scavenger, as anti‐inflammatory agent, as antioxidant, and as immune system modulator and as the cancer control agents (Hwang et al., 2006; Seelinger et al., 2008). However, p‐hydroxybenzoyl hexose, p‐coumaric acid, salicylic acid, and ellagic acid pentoside were detected from the *Z. oxyphylla* fruits. The fruites of *Z. oxyphylla* have been used for the control of anticancer activities, inhibit the growth of leukemia cell as well as ovarian cancer cell lines, breast cancer cell, and also inhibit tumor cell; they are antioxidant, antidiabetic, and anti‐inflammatory (Tang et al., 2007; Chen et al., 2004; Hwang et al., 2006; Madlener et al., 2007), and were changes in the expression of hundreds of genes in response to temperatures are followed by increases in the levels of hundreds of metabolites, some of which are known to have protective effects against the damaging effects of different stresses (Tang et al., 2007).

### 3.5 Estimation of amino acid in *Ziziphus* species

Free amino acids in plant foods have a dual role in diets. They represent a source of nitrogen and nutritionally essential amino acids such as Lys, Met, and Thr. They can also participate in reactions to form browning products. One browning product, acrylamide, formed from free Asn and glucose during food processing, is potentially toxic (Friedman and Levin, 2008; Wang et al., 2016). Here, we describe the distribution of free amino acids, and their variations in two *Ziziphus* species as enlisted in Table 7 and Figure 3. Five samples of each *Ziziphus* species were used for the estimation of amino acid by using HPLC (Agilent1000), and the free amino acid derivation method allowed to identify and quantify leucine (Lue), glycine (Gly), isoleucine (Isl), phenylalanine (Phya), valine (Val), alanine (Al), proline (Pro), and threonine (Thr), whereas (Agilent1000) amino acid analyses resulted in reliable detection and high peak resolution, concentrations, and retention time of amino acids. The highest concentration was noted of Lue 0.19 g/100 g with 596.00 retention time (RT), and Gly as 0.17 g/100 g with PA as 794.73 *Z. nummularia* sample collected from (Swat, Dir L regions) Barikot, Segrham and Ghoraghat followed by Thr>Ale>Isl>Phya> and Val except in sample Gullabad have the lowest concentration of amino acid was recorded values as 0.00 with 35.66 retention time (RT) in Phya (Table 7 and Figure 3a,b). *Z. oxyphylla* highest concentration of Luc was recorded in Kotlai collection as 0.18/100 g with 564.52 RT and Gyl 0.17 g/100 with 793.27 RT, respectively, followed by Pro>Thr>Ale>Lue>Isl>Phya> and Val except in sample Sogalai, Checkdra hill, and Gull muqam have lowest concentration of Phya was recorded values as −0.02 with 35.68 RT, 0.03 with 35.67 RT, and 0.03 with 35.74 RT, respectively, in Phya (Table 7 and Figure 3c,d).

**TABLE 7:** Free amino acid HPLC chromatogram peck area, rotation time and concentration of *Ziziphus* species

	*Z. nummularia* Swat region		*Z. nummularia* Dir (L) region		*Z. oxyphylla* Swat region		*Z. oxyphylla* Dir (L) region	
GLY								
PA	402.33	782.49	273.25	793.27	794.73	794.73	666.46	637.87
RT	14.05	13.94	14.07	14.16	14.04	14.04	13.94	14.18
g/100 g	0.09	0.16	0.06	0.17	0.17	0.17	0.14	0.13
THR								
PA	112.06	297.42	109.6	311.13	274.54	274.54	205.67	226.29
RT	15.23	15.12	15.21	15.34	15.2	15.2	15.13	15.38
g/100 g	0.03	0.11	0.03	0.11	0.1	0.1	0.07	0.08
PRO								
RT	19.61	19.54	19.6	19.7	19.6	19.6	19.55	19.77
PA	263.18	595.46	204.51	649.35	546.12	546.12	312.46	475.92
g/100 g	0.07	0.13	0.05	0.14	0.12	0.12	0.08	0.11
ALE								
RT	20.65	20.61	20.65	20.73	20.63	20.63	20.61	20.8
PA	211.34	578.57	194.96	585.49	468.02	468.02	404.57	456.3
g/100 g	0.07	0.14	0.06	0.14	0.12	0.12	0.11	0.12
VAL								
RT	30.04	30.04	30.07	30.09	30.06	30.06	30.06	30.17
PA	200.58	515.18	148.53	504.84	372.49	372.49	322.64	383.78
g/100 g	0.01	0.07	0	0.07	0.04	0.04	0.03	0.04
ISL								
RT	34.56	34.56	34.59	34.6	34.58	34.58	34.58	34.66
PA	135.01	326.84	78.97	301.08	207.83	207.83	191.69	238.93
g/100 g	0.02	0.09	−0.01	0.08	0.05	0.05	0.04	0.06
LUE								
RT	34.87	34.86	34.89	34.9	34.88	34.88	34.88	34.96
PA	229.65	596	147.42	564.52	389.33	389.33	363.47	454.08
g/100 g	0.05	0.19	0.01	0.18	0.11	0.11	0.1	0.14
PHYA								
RT	35.66	35.66	35.68	35.69	35.67	35.67	35.67	35.74
PA	86.85	212.48	36.47	172.34	113.89	113.89	142.35	156.31
g/100 g	0	0.06	−0.02	0.04	0.02	0.02	0.03	0.03

## 4 CONCLUSION

Geographical location in nature is one the most important factors that can affect the quality and quantity of a plant's material. The current findings showed that climatic conditions and soil characteristics have a major effect on the efficiency of the fruit extracts, polyphenols (TPC and TFC), antioxidant, and amount of free amino acids within the *Ziziphus* species (*Z. nummularia* and *Z. oxyphylla*). Different climatic factors as well as soil physiochemical features result in variation and indicate the intricacy of the effect of ecological as well as the availability of many chemical processes in the plant affected by these factors which cause the synthesis and or modifies the composition and thereby affect the antioxidant properties of the plant species. Overall, District Swat regions had the highest activities and the concentration of phenolic compounds (TPC and TFC), antioxidant activities (DPPH and ABTS), and the concentration of free amino acids. Fourteen metabolites from *Z. nummularia* and 13 compounds from *Z. oxyphylla* were reported based on HPLC‐DAD. However, *Z. nummularia* showed variation in concentration between them. The highest concentration of polyphenol was recorded in quercetin 3‐O‐rutinoside in both regions, while *Z. oxyphylla* had lower levels of Quercetin 3‐O‐rutinoside. Furthermore, methanolic extracts of *Ziziphus* species (*Z. nummularia* and *Z. oxyphylla*) can be proposed as an appreciated antioxidant natural source with immense utility in the development of health‐promoting nutraceuticals. Particularly, the *Z. nummularia* fruit extract shows a high level of polyphenol compound and shows good antioxidant activities. Our findings demonstrated that the *Ziziphus* species fruit extracts possess potent activities and provide great sources of natural free essential amino acids. The data also suggest that selection of *Ziziphus* species for higher phenolic and flavonoid contents or higher antioxidant potential is possible. Since the polyphenolics reported here exhibit health‐promoting properties, knowledge of both composition and concentrations of bioactive compounds of *Ziziphus* fruits can benefit consumers.

## CONFLICT OF INTEREST

The authors declare that they have no conflict of interest.

## ETHICAL APPROVAL

This article does not contain any studies involving animal's trails performed by any of the authors. Furthermore, this article does not contain any studies involving human participants performed by any of the authors.

## Supporting information

App S1

## Data Availability

The original contributions presented in the study are included in the article/Supplementary Material, and further inquiries can be directed to the corresponding author/s.
