# Supplementary material for: Distribution of polyphenolic compounds, antioxidant potential, and free amino acids in Ziziphus fruits extract; a study for determining the influence of wider geography
Source: Food Sci Nutr. 2022 Jan 28;10(5):1414–30. doi: 10.1002/fsn3.2726 (PMC9094459; doi:10.1002/fsn3.2726)
Supplement: Supplementary file 1 — App S1 [file FSN3-10-1414-s001.docx]

**Table S1** Characterization of different collected soil samples from two districts and eight different locations of KP, Pakistan in *Ziziphus* species.

| **Species** | **Texture** | **Clay (%)** | **Silt (%)** | **Sand (%)** | **PH (1:5)** | **SM** | **N (%)** | **Na (%)** | **Mg (%)** | **K (%)** |
| --- | --- | --- | --- | --- | --- | --- | --- | --- | --- | --- |
| ***Z. nummulaira* Swat region** | **Sand** | 8.8 | 20 | 71.2 | 7.6 | 34 | 0.54 ± 0.70 | 0.76 ± 0.04 | 0.45 ± 0.13 | 1.0 ± 0.20 |
|  | **Loam** | 12.8 | 48 | 39.2 | 8 | 91 | 0.89 ± 0.21 | 0.76 ± 0.60 | 0.56 ± 0.09 | 2.7 ± 0.19 |
| ***Z. oxyphylla* Swat region** | **Loam** | 16 | 47 | 36.8 | 7.4 | 89 | 0.87 ± 0.17 | 0.71 ± 0.70 | 0.45 ± 0.06 | 1.0 ± 0.03 |
|  | **Loamy sand** | 12.8 | 26 | 61.2 | 7.8 | 67 | 0.56 ± 0.90 | 0.14 ± 0.09 | 0.53 ± 0.04 | 3.0 ± 0.16 |
| ***Z. nummulaira* Dir L, region** | **Slit loam** | 14 | 63.2 | 22.8 | 7.8 | 56 | 0.67 ± 0.10 | 0.56 ± 0.09 | 0.54 ± 0.06 | 1.8 ± 0.24 |
|  | **Loam** | 12 | 43.2 | 44.8 | 7.6 | 88 | 0.48 ± 0.80 | 0.67 ± 0.06 | 0.74 ± 0.09 | 2.4 ± 0.23 |
| ***Z. oxyphylla* Dir L, region** | **Slit loam** | 18 | 67.2 | 14.8 | 7.8 | 71 | 0.15 ± 0.09 | 0.68 ± 0.22 | 0.89 ± 0.04 | 2.1 ± 0.12 |
|  |  |  |  |  |  |  |  |  |  |  |
|  | **Loam** | 14 | 57.2 | 28.8 | 7.8 | 88 | 0.47 ± 0.05 | 0.68 ± 0.09 | 0.56 ± 0.32 | 3.0 ± 0.23 |

*Note: Soil moisture (SM), Nitrogen (N), Magnesium (Mg), Sodium (Na), and potassium (K) respectively.*

**Table S2** The represented antioxidant activity of fruit methanolic extract of *Ziziphus* species *Z. nummularia* and *Z. oxyphlla*

| **Genotypes** | **Location** | **(μg/mL)** | **% ABTS** | **% DPPH** |
| --- | --- | --- | --- | --- |
|  |  |  | **Mean± SEM** | **Mean± SEM** |
| *Z. nummularia*  District  Swat | Barikot | 1000  500  250  125  62.5  31.25 | 86.54 ± 0.17  83.62 ± 0.48  76.83 ± 1.31  74.71 ± 0.94  68.71 ± 0.77  64.81 ± 1.17 | 90.05 ± 1.02  87.05 ± 0.89  80.43 ± 0.43  74.31 ± 0.54  67.29 ± 0.78  63.48 ± 1.43 |
|  | Seghram | 1000  500  250  125  62.5  31.25 | 87.67 ± 0.19  85.02 ± 0.16  78.23 ± 0.38  71.49 ± 0.42  68.78 ± 0.93  66.32 ± 0.73 | 90.89 ± 0.89  83.29 ± 0.67  80.03 ± 0.54  75.97 ± 1.12  73.70 ± 0.41  62.03 ± 0.98 |
| *Z. nummulaira*  District  Dir Lower | Ghoraghat | 1000  500  250  125  62.5  31.25 | 91.12 ± 0.73  85.15 ± 0.72  79.93 ±0.16  71.21 ± 0.28  64.34 ± 1.34  59.29 ± 0.98 | 93.60 ± 3.11  87.75 ± 0.56  79.75 ± 0.61  73.25 ± 1.05  68.23 ± 0.42  62.99 ± 0.30 |
|  | Gullabad | 1000  500  250  125  62.5  31.25 | 92.11 ± 0.83  89.36 ± 0.71  80.41 ± 0.77  71.21 ± 0.37  65.11 ± 0.98  60.17 ± 0.44 | 91.89 ± 1.09  86.09 ± 0.13  81.98 ± 1.05  73.72 ± 0.16  68.27 ± 0.17  62.26 ± 0.34 |
| *Z. oxyphylla*  District  Swat | Kotlai | 1000  500  250  125  62.5  31.25 | 91.03 ± 0.36  87.34 ± 0.34  81.43 ± 0.43  76.51 ± 0.61  67.19 ± 0.77  61.55 ± 0.77 | 91.83 ± 0.98  86.12 ± 0.14  79.98 ± 1.01  73.71 ± 0.13  64.29 ± 1.03  60.66 ± 0.56 |
|  | Sogalai | 1000  500  250  125  62.5  31.25 | 89.58 ± 0.44  85.34 ± 0.21  79.16 ± 0.42  72.51 ± 0.61  65.19 ± 0.87  60.45 ± 0.78 | 89.68 ± 0.96  82.67 ± 0.14  75.34 ± 1.21  68.43 ± 0.89  63.11 ± 0.18  59.66 ± 0.21 |
| *Z. oxyphylla*  District  Dir Lower | Checkdra hill | 1000  500  250  125  62.5  31.25 | 89.21 ± 0.53  84.14 ± 0.70  76.93 ±0.62  71.21 ± 0.28  67.33 ± 0.89  59.09 ± 1.19 | 93.60 ± 3.11  87.75 ± 0.56  87.75 ± 0.56  73.25 ± 1.05  68.23 ± 0.42  62.99 ± 0.30 |
|  | Gull muqam | 1000  500  250  125  62.5  31.25 | 88.22 ± 0.73  83.46 ± 0.75  79.50 ± 0.77  72.00 ± 0.67  64.76 ± 0.98  59.28 ± 0.44 | 89.09 ± 1.08  86.13 ± 0.17  80.89 ± 1.07  74.62 ± 0.24  69.17 ± 0.20  63.24 ± 0.46 |
| Ascorbic acid |  | 1000  500  250  125  62.5  31.25 | 95.32 ± 1.54  89.54 ± 0.47  82.36 ± 0.44  76.91 ± 0.49  71.74 ± 0.82  67.61 ± 0.71 | 97.77 ± 0.28  91.67 ± 0.72  85.96 ± 0.29  80.10 ± 0.57  73.48 ± 0.76  70.70 ± 0.47 |

*Data are calculated as mean ± standard deviation (n = 3). Values in the same column with different superscripts*

*are significantly different (P < 0.05)*


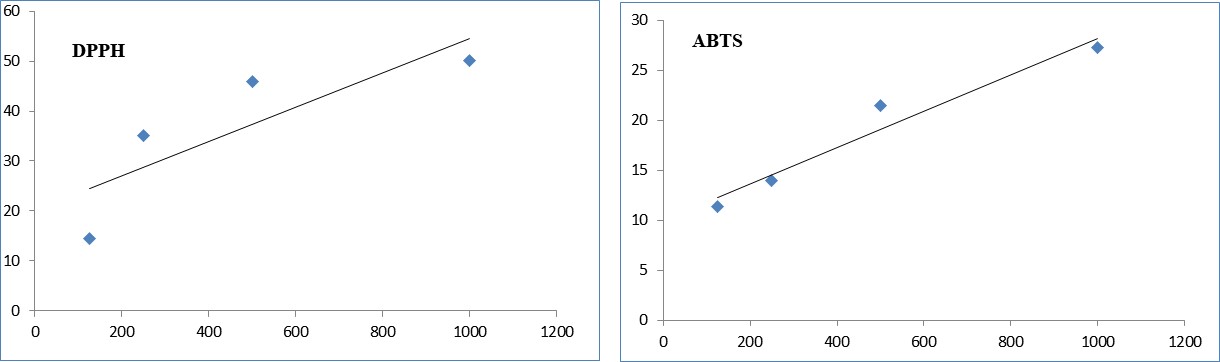


**Figure S1** The correlation between DPPH and ABTS free radical scavenging activities of the *Ziziphus* species
